# Nitrobenzoate-Derived Compound X8 Impairs Vascular Development in Zebrafish

**DOI:** 10.3390/ijms23147788

**Published:** 2022-07-14

**Authors:** Chien-Chih Chiu, Hsieng-Kuo Chin, Sen-Yuan Chung, Kuan-Hsuan Hsieh, Yi-Shan Huang, Mei-Feng Huang, Yi-Hao Lo, Zhi-Hong Wen, Chang-Yi Wu

**Affiliations:** 1 Department of Biological Sciences, National Sun Yat-sen University, Kaohsiung 804, Taiwan; cchiu@kmu.edu.tw (C.-C.C.); slime4172@mail.kmsh.tn.edu.tw (S.-Y.C.); c3493467@hotmail.com (K.-H.H.); a0928020114@gmail.com (Y.-S.H.); fengerl@mail.ncku.edu.tw (M.-F.H.); 2 Department of Biotechnology, Kaohsiung Medical University, Kaohsiung 807, Taiwan; 3 Department of Medical Research, Kaohsiung Medical University Hospital, Kaohsiung 807, Taiwan; 4 Department of Surgery, Kaohsiung Armed Forces General Hospital, Kaohsiung 802, Taiwan; cvschin@gmail.com; 5 Department of Marine Biotechnology and Resources, National Sun Yat-sen University, Kaohsiung 804, Taiwan; loveangelsome@yahoo.com.tw (Y.-H.L.); wzh@mail.nsysu.edu.tw (Z.-H.W.); 6 Institute of Medical Science and Technology, National Sun Yat-sen University, Kaohsiung 804, Taiwan; 7 Doctoral Degree Program in Marine Biotechnology, National Sun Yat-sen University, Kaohsiung 804, Taiwan; 8 Department of Physiology, College of Medicine, National Cheng Kung University, Tainan 701, Taiwan; 9 Department of Family Medicine, Zouying Branch of Kaohsiung Armed Forces General Hospital, Kaohsiung 802, Taiwan

**Keywords:** nitrobenzoate compound, intersegmental vessel, caudal vein plexus, vascular development and zebrafish

## Abstract

Proper growth and patterning of blood vessels are critical for embryogenesis. Chemicals or environmental hormones may interfere with vascular growth and cause developmental defects. Nitrobenzoate-based compounds have been demonstrated to have a wide range of biological and pharmacological functions, leading to the development of numerous 4-nitrobenzoate derivatives for clinical application. In this study, we tested a novel nitrobenzoate-derived compound, X8, and investigated its effects on vascular development using zebrafish as a model organism. We first determined the survival rate of embryos after the addition of exogenous X8 (0.5, 1, 3, 5, and 10 μM) to the fish medium and determined a sublethal dose of 3 μM for use in further assays. We used transgenic fish to examine the effects of X8 treatment on vascular development. At 25–32 h postfertilization (hpf), X8 treatment impaired the growth of intersegmental vessels (ISVs) and caudal vein plexuses (CVPs). Moreover, X8-treated embryos exhibited pericardial edema and circulatory defects at 60–72 hpf, suggesting the effects of X8 in vasculature. Apoptosis tests showed that the vascular defects were likely caused by the inhibition of proliferation and migration. To investigate the molecular impacts underlying the defects in the vasculature of X8-treated fish, the expression levels of vascular markers, including *ephrinb2*, *mrc1*, and *stabilin*, were assessed, and the decreased expression of those genes was detected, indicating that X8 inhibited the expression of vascular genes. Finally, we showed that X8 treatment disrupted exogenous GS4012-induced angiogenesis in *Tg(flk:egfp)* zebrafish embryos. In addition, vascular defects were enhanced during cotreatment with X8 and the VEGFR2 inhibitor SU5416, suggesting that X8 treatment causes vascular defects mediated by disruption of VEGF/VEGFR2 signaling. Collectively, our findings indicate that X8 could be developed as a novel antiangiogenic agent.

## 1. Introduction

The establishment of a blood vessel network in vertebrates is critical for embryonic survival and requires precise patterning and regulation [1]. The two main processes in blood vessel formation are called vasculogenesis and angiogenesis. Arteries and veins are formed and specified from angioblast progenitor cells during vasculogenesis [2,3]. Angiogenesis is the process of new blood vessels sprouting from preexisting vessels and growing into a vascular network after the creation of arteries and veins [4]. Because of the optical transparency of its embryos and the availability of genetic tools, the zebrafish (*Danio rerio*) is an excellent model for studying vascular development [5]. Furthermore, evolutionarily conserved molecular processes driving vascular formation in invertebrates have been discovered. Many successful studies addressing the molecular mechanisms of blood vessel development, such as angioblast specification and migration, vascular endothelium proliferation and migration, and vessel formation and patterning, have been conducted using zebrafish as a model organism [5,6,7,8,9,10,11]. Furthermore, the effects of chemicals and drugs on the development of vasculature have been successfully addressed in the zebrafish model [12,13,14,15,16]. For example, we reported that the marine compound WA-25 inhibits angiogenesis [13], GB9 impairs vascular development [14], and heteronemin (SP1) suppresses lymphangiogenesis [15]. Moreover, the poly-naphthol compound ST104T [12] and the peptide hormone α-melanocyte-stimulating hormone (α-MSH) suppress angiogenesis [16]. The related findings will enhance our ability to treat human vascular diseases via genetic and pharmaceutical approaches.

The differentiation and specification of arteries and veins from angioblast progenitors are mainly regulated by the VEGF/Notch signaling pathways [2,9]. After the formation of the main vessels, endothelial progenitor cells further proliferate and migrate to form a patterned vessel network. The rapid growth and patterning of the intersegmental vessel (ISV) in the zebrafish trunk provides an ideal window to study angiogenesis. Many novel genetic molecules have been identified that participate in regulating dorsal sprouting and migration to form ISVs via VEGF/VEGFR2 signals. Furthermore, endothelial cells sprout ventrally at the tail region to form a honeycomb-like vessel network called the caudal vein plexus (CVP), which is mediated by BMP signaling pathways [17,18]. Knowledge of vascular patterning and the molecular control of vascular development in zebrafish provides a useful platform for evaluating and screening the effects of chemicals or drugs on vascular growth.

Nitrobenzoate-based compounds have been reported to exert various biological activities, including antimalarial [19], antibiotic [20], and anticancer [21,22] activities. Nitrobenzoate-based compounds have been reported to suppress metastatic activity by inhibiting the mucin-type, transmembrane glycoprotein, podoplanin-stimulated tumor-cell-induced platelet aggregation [22] and inhibit multidrug-resistant cancer by inhibiting tubulin polymerization [23]. Although some biological activities of nitrobenzoate-derived compounds have been studied, the angiogenesis-related biological activity of nitrobenzoate-derived compounds is much less studied. Accordingly, the study of nitrobenzoate derivatives to enhance antitumor and antiangiogenic activity and reduce side effects could be a promising strategy. Thus, in this study, we tested a novel nitrobenzoate-derived compound, X8, and investigated its effects on vascular development.

Here, we showed a reduction in ISV growth and a decrease in the sprouting and formation of CVP endothelial cells in embryos treated with the nitrobenzoate-derived compound X8. We further showed that the vascular defects were due to the impairment of endothelial cell migration and proliferation. Consistent with the defects, X8 treatment resulted in decreased expression of vascular markers. Finally, we showed that X8 treatment caused vascular defects mediated by disruption of VEGF/VEGFR2 signaling. Together, our study indicates that X8 could be developed as a novel antiangiogenic agent.

## 2. Results

### 2.1. X8 Treatment Impairs Vascular Development during Zebrafish Embryogenesis

**Figure 1:**
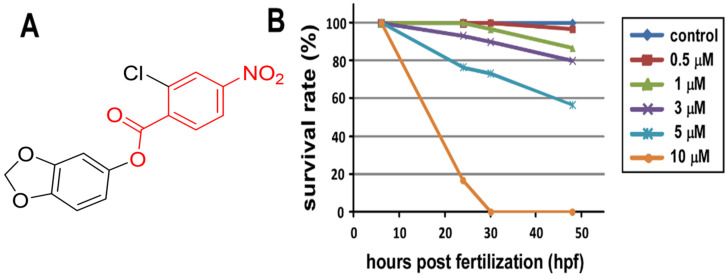
**Chemical structure of X8 and toxic effects of X8 on zebrafish embryos**. (**A**) The chemical structure of the nitrobe*n*zoate-derived compound X8 (2*H*-13-benzodioxol-5-yl 2-chloro-4-nitrobenzoate) and the molecular weight is 321.7 g/mol. The red color part indicates a nitrobenzoate structure. (**B**) The embryos of wild-type zebrafish were treated (n = 30) with the indicated concentrations of X8 (from 0.5 to 10 μM) at 6 hpf, and survival rates at 24, 30, and 48 hpf were recorded. The survival rate at 6 hpf was defined as 100%.

We were interested in characterizing the genetic control of vascular development and identifying novel molecules with proangiogenic or antiangiogenic effects. Thus, we analyzed the effects of treatment with the nitrobenzoate-derived compound X8 (2*H*-13-benzodioxol-5-yl 2-chloro-4-nitrobenzoate, Figure 1A) on vascular function using zebrafish as a model. To assess the vascular effects of X8, we first examined its toxic effects on zebrafish embryos. Treatment with 10 μM X8 caused a high mortality rate (~80%) with an aberrant morphology at 48 h postfertilization (hpf). However, embryos treated with 1–5 μM X8 had a survival rate of more than 60%, without an aberrant fish shape. Thus, we used 3 μM X8 for further experiments (Figure 1B).

**Figure 2:**
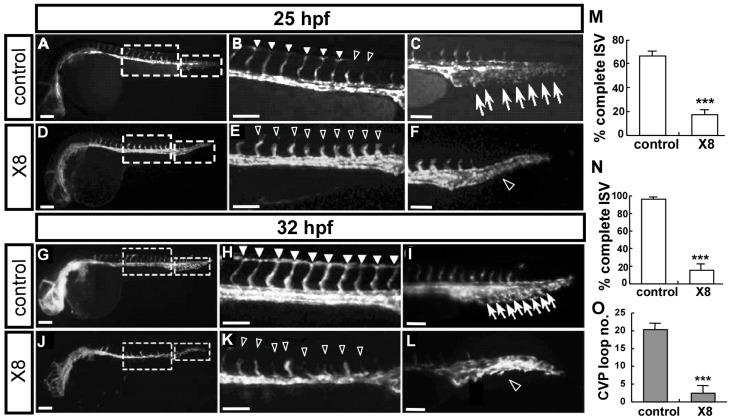
**X8 treatment impairs vascular development during zebrafish embryogenesis.** (**A**–**F**) Lateral view of X8-treated *Tg(flk:eGFP)* embryos at 25 hpf (**D**) showing decreased ISV growth to the top of the embryo (hollow arrowheads in (**E**)) and decreased or a complete absence of angiogenic sprouting from the caudal vein (hollow arrowhead in (**F**)) compared with those parameters in wild-type control embryos ((**A**), arrowheads in (**B**), and arrows in (**C**)). (**A**,**D**) Fluorescent images of whole larvae; (**B**,**E**) and (**C**,**F**) are enlargements of the truck and tail regions of (**A**,**D**). (**G**–**L**) At 32 hpf, in untreated control embryos, ISVs reached the DLAV ((**G**) and arrowheads in (**H**)), and the CVP formed honeycomb-like loop structures at the tail ((**I**), arrows). Meanwhile, ISVs were stalled in mid-somite in X8-treated embryos ((**J**) and hollow arrowheads in (**K**)), and decreased endothelial cell sprouting and loop formation at the CVP (hollow arrowhead in (**L**)) were observed. (**H**,**I**) are enlargements of the truck and tail regions of (**G**) and (**K**,**L**) are enlargements from (**G**). (**M**) Quantification of the percentage of complete ISV structures showed a 3.5-fold decrease in X8-treated fish at 25 hpf. (**N**,**O**) Quantification of the percentages of complete ISV and CVP structures in X8-treated fish at 32 hpf showed reductions of ~5.2-fold and ~6-fold, respectively (n = 25 for control and n = 27 for X8 samples). *** *p* < 0.0001 by unpaired Student’s *t*-test. Scale bars: 200 μm in (**A**,**D**,**G**,**J**); 100 μm in (**B**,**C**,**E**,**F**,**H**,**I**,**K**,**L**).

At 25 hpf, in transgenic *Tg(flk:eGFP)* embryos with endothelial-cell-specific GFP expression, treatment with 3 μM X8 resulted in decreased ISV growth at the top of the embryo and decreased or a complete lack of angiogenic sprouting from the caudal vein (Figure 2E,F) relative to these parameters in wild-type control embryos (Figure 2B,C,M). At 32 hpf, in control embryos, ISVs reached the dorsal region of the embryo and formed the dorsal longitudinal anastomotic vessel (DLAV), and the CVP formed loop network structures at the tail (Figure 2H,I). At the same stage, ISVs nearly stopped growth at mid-somite in X8-treated embryos with an ~95% uncompleted ISV structure and a decrease of ~6-fold in CVP loops at the tail area compared with wt control embryos (Figure 2K,L,N,O). These data suggest that X8 treatment inhibits vascular growth during zebrafish development.

### 2.2. X8 Treatment of Embryos Results in Pericardial Edema and Circulatory Defects

**Figure 3:**
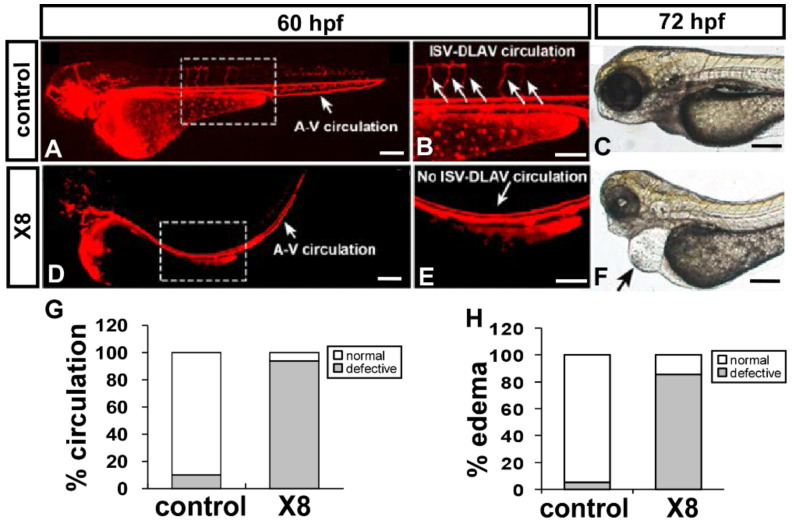
**X8 treatment of embryos results in pericardial edema and circulatory defects.** (**A**,**B**,**D**,**E**) X8-treated transgenic *Tg(gata1:dsRed*) embryos at 60 hpf. X8-treated fish showed circulatory defects (**D**,**E**) compared with wild-type fish (**A**,**B**). (**G**) Circulatory defects at the ISV-DLAV part and slow to no axial circulation in the aorta/vein in the trunk region were quantitated in DMSO control (n = 24) and X8-treated embryos (n = 22) at 60 hpf. (**C**,**F**) A representative image of edema in a fish (arrow in (**F**)) at 72 hpf is shown. (**H**) Quantitative analysis showed that 82% of X8-treated embryos (n = 20) exhibited mild–severe pericardial edema relative to control embryos (n = 21). Scale bars: 200 μm in (**A**,**C**,**D**,**F**); 100 μm in (**B**,**E**).

We observed vascular growth defects at 25–32 hpf in X8-treated embryos. It has been reported that edema and lack of circulation are side effects of vascular malformation; therefore, we first examined the blood flow in wild-type zebrafish embryos and X8-treated transgenic *Tg(gata1:dsRed)* embryos with blood cell-specific dsRed fluorescent expression. At 48–72 hpf, X8 treatment caused an increase in pericardial edema and circulatory defects. In addition, ~93% of X8-treated embryos exhibited slow to no circulation in the primary axial vessels and/or at the ISV-DLAV part at 60 hpf (n = 24 for control and n = 22 for X8-treated embryos) (Figure 3A,B,D,E,G). At 72 hpf, ~82% of embryos showed pericardial edema, a higher percentage than that of wt control embryos (5%) (Figure 3C,F,H). These data were correlated with the vasculature defects in X8-treated fish.

### 2.3. X8 Treatment Inhibits the Growth of ISV Cells

**Figure 4:**
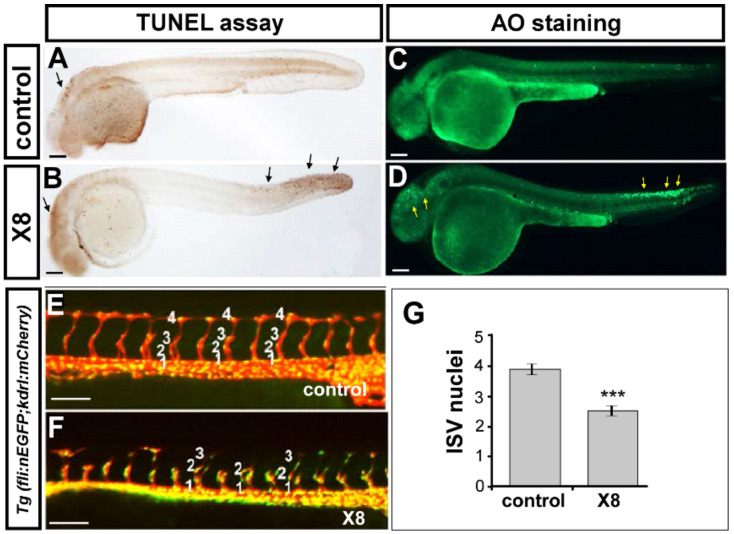
**X8 treatment inhibited the growth of ISV cells.** (**A**–**D**) A TUNEL assay and AO staining were used to assay apoptotic cells in wild-type (**A**,**C**) and X8-treated embryos (**B**,**D**) at 30 hpf. An increase in apoptotic cells was observed close to the head and tail regions in X8-treated embryos compared with untreated control embryos (**B**,**D**), but no increase was observed in vascular regions (**E**,**F**). The number of cells forming each ISV was counted in control (**E**) and X8-treated (**F**) *Tg(kdrl:mCherry; fli1a:negfp)^y7^* embryos at 30 hpf. (**G**) Average number of endothelial cells per ISV: 3.9 ± 0.4 (n = 80) and 2.5 ± 0.4 (n = 60) in wild-type and X8-treated embryos, respectively. *** *p* < 0.0001 by unpaired Student’s *t*-test. Scale bars: 100 μm in (**A**–**F**).

The vascular abnormalities caused by X8 treatment could be due to a decrease in endothelial cell proliferation and migration or an increase in cell apoptosis. To test these hypotheses, we first examined whether cell death was increased by X8 treatment. We performed acridine orange (AO) staining and a TdT-mediated dUTP-X nick-end labeling (TUNEL) assay at 30 hpf and found that an increase in apoptotic cells were observed close to the head and tail regions but not in vascular regions in X8-treated embryos compared with untreated control embryos (Figure 4A–D), suggesting that the vascular flaws may not be caused by endothelial cell death. To determine whether X8 treatment decreases cell migration or proliferation, we examined ISV growth and the number of endothelial cells per ISV using transgenic *Tg(kdrl:mCherry; fli1a:negfp ^y7^)* fish, in which GFP is expressed in the nucleus of endothelial cells and mCherry is expressed on ISV endothelial cells. At 30 hpf, compared with untreated wild-type control embryos, X8-treated embryos showed slow or stopped growth at the middle of somites and a significantly reduced number of ISV cells (Figure 4E–G). These data suggest that X8 culture impairs ISV cell growth, likely disrupting the proliferation and migration of endothelial cells.

### 2.4. X8 Treatment Reduces the Expression Levels of Vascular-Specific Markers

**Figure 5:**
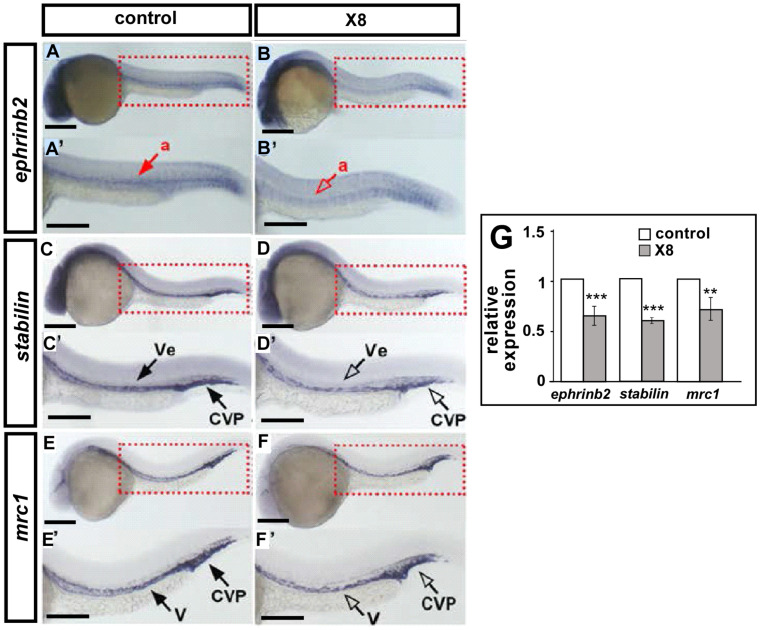
**X8 treatment of embryos decreases the expression of vascular markers.** (**A**–**F**,**A’**–**F’**) Compared with wild-type control embryos (**A**,**C**,**E**), X8-treated embryos exhibited decreased expression of the arterial (a) marker *ephrinb2* (**B**), the panvascular (v) marker *stabilin* (**C**), and the venous (ve) marker *mrc1* (**E**) in the trunk vessels and CVP at 24 hpf. (**G**) Quantification of the relative expression level by qPCR showed reductions of approximately 30–40% in the expression of the vascular markers *ephrinb2* (0.6 ± 0.15), *stabilin* (0.6 ± 0.1), and *mrc1* (0.7 ± 0.16) in X8-treated embryos. *** *p* < 0.0001 and ** *p* < 0.001 by unpaired Student’s *t*-test. The data are presented as the mean ± SD values. Scale bars: 200 μm in (**A**–**H**).

The vascular defects in the growth of ISV and the formation of CVP suggested that X8 treatment affects important processes in vascular development. Therefore, we analyzed the expression levels of vascular markers at 24 hpf by whole-mount in situ hybridization and quantitative PCR (qPCR). The expression levels of the arterial marker *ephrinb2*, venous marker *mrc1*, and panvascular marker *stabilin* were decreased in the trunk and CVP of X8-treated embryos compared with wild-type embryos (Figure 5A–F,A’–F’). The decreased expression levels of vascular markers were also quantified by quantitative PCR (qPCR), and a significant 30–40% decrease in expression was found in X8-treated embryos (Figure 5G). These results suggest that X8 treatment downregulates the expression of vascular-associated genes to attenuate vessel formation.

### 2.5. Interaction between X8 and VEGF Signaling

**Figure 6:**
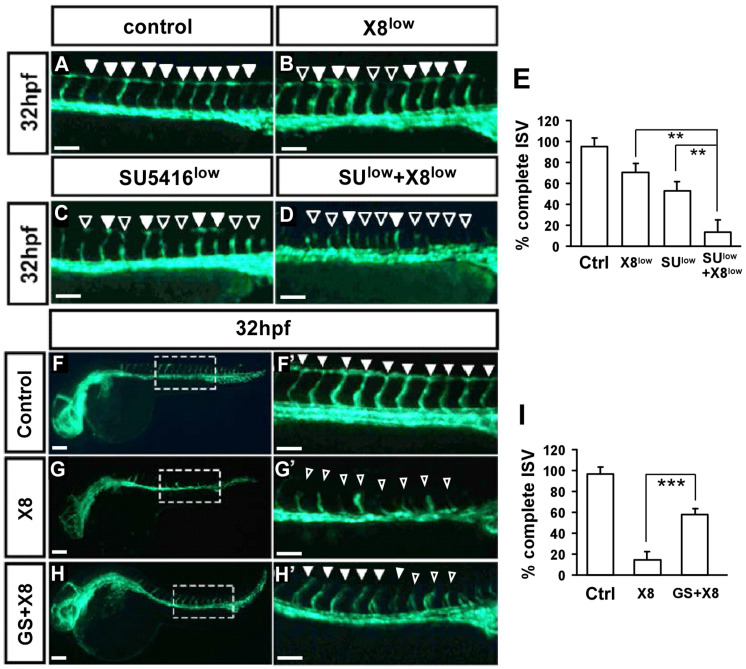
**Interaction between X8 and VEGF signaling.** (**A**–**E**) Enhanced effects of vascular defects in embryos cotreated with X8 and SU5416. At 32 hpf, in untreated control embryos (**A**), ISVs reached the DLAV (*arrowheads*). ISV growth was weakly affected in embryos treated with a lower concentration of X8 (1.5 μM) (**B**) or a lower concentration of SU5416 (5 μM) (**C**). Embryos treated with the combination of X8 and SU5416 showed enhanced vascular defects in ISV growth (hollow arrowheads in (**D**)). (**E**) The quantitative analysis of ISV structures showed an enhanced reduction in X8- and SU5416-cotreated embryos (n = 20) at 32 hpf. (**F**–**H**,**F’**–**H’**) Rescue effect of the angiogenic compound GS4012 in X8-treated *Tg(flk:egfp)* zebrafish embryos. Embryos were treated with X8 (3 μM) in combination with (**H**,**H’**) or without (**G**,**G’**) GS4012 (1 μg/mL) at 6 hpf and were then imaged at 32 hpf. The percentage of complete ISV structures is quantified in (**I**). The data are presented as the mean ± SD values (n = 20). The asterisks indicate statistical significance versus control embryos. ** *p* < 0.001 and *** *p* < 0.0001 by unpaired Student’s *t*-test. Scale bars: 100 μm in (**A**–**D**,**F’**–**H’**); 200 μm in (**F**–**H**).

We showed that X8 treatment of embryos impaired ISV growth, accompanied by a reduction in ISV endothelial cells. Additionally, X8 treatment caused defects in CVP formation. VEGF/VEGFR2 signals are essential for vascular development during ISV angiogenesis. Thus, we hypothesized that X8 impairs vascular development mediated by VEGF signaling. To test this hypothesis, we altered VEGF signals by using the VEGFR2 inhibitor SU5416 [24] and the proangiogenic compound GS4012. We investigated whether any effects on embryos were enhanced by combined treatment with lower concentrations of X8 and lower concentrations of SU5416. Embryos cotreated with SU5416 and X8 were more susceptible to defects in ISV growth than those treated with either single agent at 32 hpf (Figure 6A–E). On the other hand, we demonstrated that the vascular defects in X8-treated *Tg(flk:egfp)* zebrafish embryos were rescued by exogenous treatment with the angiogenesis inducer GS4012 (Figure 6F–I). Collectively, these data suggest that X8 treatment causes vascular defects mediated by the interaction of X8 with VEGF/VEGFR2 signaling.

## 3. Discussion

Nitrobenzoate-derived compounds have been studied in inflammation suppression and cancer treatment. Thus, they may serve as useful chemicals for new agents to treat inflammatory diseases and/or cancer. Although the anticancer effects of nitrobenzoate-derived compounds are well-documented, most studies have focused on nitrobenzoate-compound-induced proliferation inhibition and apoptosis induction in cancer cells, and the angiogenesis-related biological activity of nitrobenzoate derivatives is much less studied. Recently, the synthetic nitrobenzoate compound IMB5476 (2-morpholin-4-yl-5-nitro-benzoic acid 4-methylsulfanyl-benzyl ester) was reported to exert a potent in vitro antiangiogenic effect targeting endothelial cell mobility and tube formation of HMEC-1 cells [25]. However, the underlying mechanism of nitrobenzoate-induced anti-angiogenesis and further in vivo investigation are still needed. In this study, we analyzed the effects of X8 treatment on vascular function using zebrafish as an in vivo model. In X8-treated embryos, we found a decrease in ISV cells as well as a reduction in the sprouting and loop structures of CVP endothelial cells, indicating decreased cell proliferation and migration. In X8-treated embryos, decreased expression of vascular markers was observed, which corresponded to vascular abnormalities. Finally, we showed that X8 disrupts angiogenesis, an effect likely mediated by interaction with VEGF/VEGFR2 signaling. Collectively, our study provides a useful platform to examine vascular growth during chemical treatment.

We have screened for novel potential antiangiogenic drugs for many years [12,13,14]. Interestingly, X8 is the first nitrobenzoate compound examined that exerts bioactivity related to anti-angiogenesis in our laboratory. More importantly, since many nitrobenzoate compounds have shown anti-inflammatory and anticancer activities, X8 will be tested to determine whether it exhibits the above activities. Our study indicates that X8 can likely be developed for antiangiogenic therapy, for example, to inhibit tumor- or diabetes-related angiogenesis. Thus, the potent antiangiogenic effect and low toxicity of X8 may have the potential for drug development for angiogenesis-related diseases.

Our results showed that X8 treatment causes vascular deficiencies during embryogenesis, which are likely mediated by VEGF signaling. The underlying molecular mechanism, for example, the target genes of X8, remains unclear. Our results revealed that X8 treatment may interact with VEGFR2, a protein that has been shown to mediate most VEGF-A signaling in endothelial cells, resulting in cell differentiation, migration, proliferation, vascular permeability, vascular patterning, and the angiogenic response. As a result, interfering with the VEGF signaling pathway is thought to be a potential approach through which X8 mediates antiangiogenic effects. Moreover, BMP signaling has been shown to be critical for CVP patterning and it will be interesting to test whether it interacts with X8. Thus, in the near future, cell-based research will be conducted to confirm the antivascular effects of X8 and to identify the interactions between VEGF or the BMP signaling pathway and X8 that mediate angiogenesis inhibition, which will benefit clinical applications for angiogenesis-related diseases.

## 4. Materials and Methods

### 4.1. Zebrafish and Husbandry

Wild-type AB strain zebrafish and the transgenic lines *Tg*(*kdrl*:*eGFP*)*^la116^*, *Tg*(*kdrl*:*mCherry*)*^ci5^*, *Tg*(*gata1*:*dsRed*), and *Tg*(*fli1a*:*negfp*)*^y7^* [26,27,28,29,30] obtained from the Taiwan Zebrafish Core Facility were raised and maintained in a 28.5 °C fish room. The National Sun Yat-sen University Institution Animal Care and Use Committee approved all experimental procedures, which were carried out in compliance with them (IACUC approval no. 10720).

### 4.2. Zebrafish Embryo and Chemical Treatments

The chorion was removed from zebrafish embryos grown in E3 fish media by incubation in 20 mg/mL pronase (Sigma, St. Louis, MO, USA). According to Kimmel’s work [31], embryos were staged in hours postfertilization (hpf), and pigmentation was suppressed by adding 0.003% 1-phenyl-2-thiourea (PTU; Sigma) at approximately 6 hpf. Treatment with the nitrobenzoate-derived compound X8 (Enamine Ltd. Monmouth Jct., NJ, USA), SU5416 (Calbiochem, Nottingham, UK), and GS4012 (Sigma) was performed with fresh stock. Stock solutions of 10 mg/mL X8, 10 mM SU5416, and 10 mg/mL GS4012 were prepared in 0.3% dimethyl sulfoxide (DMSO) (Sigma) and diluted to the working concentrations. All chemicals were added to the E3 medium at 6 hpf to prevent the disruption of early development.

### 4.3. RNA Extraction and cDNA Preparation

Total RNA of the embryos from the indicated stages of embryonic development was extracted using the RNeasy Kit (Qiagen, Valencia, CA, USA) according to the manufacturer’s instructions. In brief, 1 µg of total RNA, oligo(dT) primers (Invitrogen, Philadelphia, PA, USA), and RT-reverse transcriptase (Roche, Branford, CT, USA) were used to synthesize first-strand cDNA according to the manufacturer’s instructions.

### 4.4. Real-Time Quantitative PCR (RT–qPCR)

A LightCycler 96 Real-Time PCR Detection System (Roche) and SYBR Green I Master Mix were used for performing the real-time PCR assay. The NCBI Primer-BLAST tool was used to design the sequences of the PCR primers (Table 1). The Ct values of gene expression were measured by Roche LC96 software. Relative expression levels were calculated by using the ΔΔCt method, in which the Ct value of each gene was normalized to the level of the reference gene β-actin (ΔCt) and then normalized to untreated control samples (ΔΔCt) to generate relative expression. The triplicates were used for all reactions.

### 4.5. Whole-Mount In Situ Hybridization

Digoxigenin (DIG)-labeled antisense riboprobes were made for each gene by T7 Polymerase (Roche) and a DIG RNA labeling kit (Roche) according to the manufacturer’s instructions. A high-resolution in situ hybridization protocol was performed based on Thisse et al. and previous publications [32,33]. The sequence and probe generation of *ephrinb2*, *mrc1*, and *stabilin* have been disclosed in previous studies [34,35]. In short, embryos were preserved with 4% paraformaldehyde (PFA) and kept in methanol at −20 °C to dehydrate. Samples were rehydrated in phosphate-buffered saline with 0.1% Tween-20 (PBST) and then partially digested with proteinase K. Embryos were incubated overnight at 70 °C with the RNA probe in a 50% formamide-based hybridization solution. After the washing and blocking processes, an anti-Dig conjugated with alkaline phosphatase antibody was added to the embryos and incubated overnight at 4 °C. Excess antibody was removed by washes in PBST and color reaction with the NBT/BCIP substrate (Roche). The stained embryos were embedded and imaged with a microscope.

### 4.6. Imaging and Data Processing

Embryos were mounted with either 1.5% low-melting-point agarose (Invitrogen) or 3% methylcellulose (Sigma), and the images were acquired with a color digital AxioCam HRc camera (Carl Zeiss, Jena, Germany) or a SPOT RT3 camera (Diagnostic Inc., Sterling heights, MI, USA). For confocal imaging, embryos were embedded with 5% tricaine, and images were acquired with a Zeiss LSM700 or Nikon Eclipse 90i C1 confocal microscope and processed with ImageJ software (NIH, Bethesda, MD, USA).

### 4.7. AO Staining

We dechorionated the embryos and inoculated them in E3 fish medium with 2 µg/mL AO. After 30 min, these embryos were further mounted and then photographed under a Carl Zeiss Lumar V12 stereomicroscope fitted with a color digital AxioCam HRc camera after washing with E3 media (Carl Zeiss).

### 4.8. TUNEL Assay

A transferase-mediated dUTP-digoxigenin nick-end labeling (TUNEL) assay was conducted according to the manufacturer’s instructions using an in situ cell death detection kit (Roche). In a nutshell, embryos were preserved and kept in methanol. After rehydration and permeabilization with Proteinase K (Roche), the embryos were refixed with 4% PFA for 15 min, treated with 3% H_2_O_2_ to quench endogenous peroxidase (POD) activity, and then incubated for 3 h at 37 °C in the dark with 45 µL of TUNEL solution plus 5 µL of TUNEL enzyme. The embryos were then rinsed in PBS, blocked in PBS with 5% serum (Thermo Scientific, Waltham, MA USA), incubated overnight in the dark with a POD-conjugated anti-fluorescein antibody (Roche), washed with PBS buffer, and visualized using DAB as a substrate.

## Figures and Tables

**Table 1:** Quantitative PCR (qPCR) primer sequences used in this study.

qPCR Primer Name	Sequence
*β-actin*_qf	5′-CTCTTCCAGCCTTCCTTCCT-3′
*β-actin*_qr	5′-CTTCTGCATACGGTCAGCAA-3′
*mrc1*_qf	5′-CTAGCAAGCCTGAAGGTGCC-3′
*mrc1*_qr	5′-TGAGAGGCTGGGTAGTTGGG-3′
*ephrinb2*_qf	5′-CTGGAACACCACGAACACC-3′
*ephrinb2*_qr	5′-CACACGTGGGCAAACTATGT-3′
*stabilin*_qf	5′- GGGCTTCCAATACCAACTGG -3′
*stabilin*_qr	5′- CCTGGTTGCACAGACAGACC -3′

## Data Availability

Not applicable.
